# Supplementary material for: Alum Pickering Emulsion as Effective Adjuvant to Improve Malaria Vaccine Efficacy
Source: Vaccines (Basel). 2021 Oct 26;9(11):1244. doi: 10.3390/vaccines9111244 (PMC8624716; doi:10.3390/vaccines9111244)
Supplement: Supplementary file 1 [file vaccines-09-01244-s001.zip › vaccines-1235401-supplementary.pdf]

Supplemental data

Table S1. Serum biochemical parameters (*n* = 8).

Group	BUN (U/L)	ALT (U/L)	AST (U/L)	LDH (U/L)	ALP (U/L)
PBS	7.9 ± 0.4	31.6 ± 5.8	73.1 ± 11.2	1683.5 ± 374.5	95.0 ± 2.9
Al	8.0 ± 0.4	33.4 ± 6.2	77.9 ± 15.4	1550.5 ± 424.2	109.8 ± 11.9
ALPE	7.3 ± 0.7	33.9 ± 4.8	70.0 ± 11.1	1282.0 ± 221.4	97.0 ± 20.5
ALMPE	7.4 ± 0.4	34.0 ± 2.4	76.8 ± 5.7	1302.3 ± 125.3	93.6 ± 5.5
